# Silica nanoparticle stability in biological media revisited

**DOI:** 10.1038/s41598-017-18502-8

**Published:** 2018-01-09

**Authors:** Seon-Ah Yang, Sungmoon Choi, Seon Mi Jeon, Junhua Yu

**Affiliations:** Department of Chemistry and Education, Seoul National University, 1 Gwanak-Ro, Gwanak-Gu, Seoul, 08826 South Korea

## Abstract

The stability of silica nanostructure in the core-silica shell nanomaterials is critical to understanding the activity of these nanomaterials since the exposure of core materials due to the poor stability of silica may cause misinterpretation of experiments, but unfortunately reports on the stability of silica have been inconsistent. Here, we show that luminescent silver nanodots (AgNDs) can be used to monitor the stability of silica nanostructures. Though relatively stable in water and phosphate buffered saline, silica nanoparticles are eroded by biological media, leading to the exposure of AgNDs from AgND@SiO_2_ nanoparticles and the quenching of nanodot luminescence. Our results reveal that a synergistic effect of organic compounds, particularly the amino groups, accelerates the erosion. Our work indicates that silica nanostructures are vulnerable to cellular medium and it may be possible to tune the release of drug molecules from silica-based drug delivery vehicles through controlled erosion.

## Introduction

Multifunctional nanomaterials have attracted considerable attention, particularly in the field of drug delivery^1,2^. Thanks to their controllable syntheses, silica nanostructures are frequently used as building blocks of the above nanomaterials to form core-shell or mesoporous structures^3–7^. In addition to their outstanding biocompatibility, the silica shells significantly improve the stability of the protected substances in the cores, while interfering negligibly with their chemical and physical properties^8–14^. The advantages of silica have prompted the development of an enormous amount of biological applications for silica nanostructures, where they mainly act as protection layers or drug delivery vehicles, which assumes that silica in biological media is highly stable^15–18^. However, it is well known that water can dissolve silica^19,20^. However, reports on the stability of silica have been inconsistent^1,21–24^. Since, when mostly silica nanostructures are exposed to a biological medium, the stability of silica nanostructures and the interaction between silica and such media may determine the activity of bionanomaterials *in vivo*. There is a pressing need for a clear understanding of silica stability in biological media due to the wide biological applications of silica nanostructures^25^.

**Figure 1:**
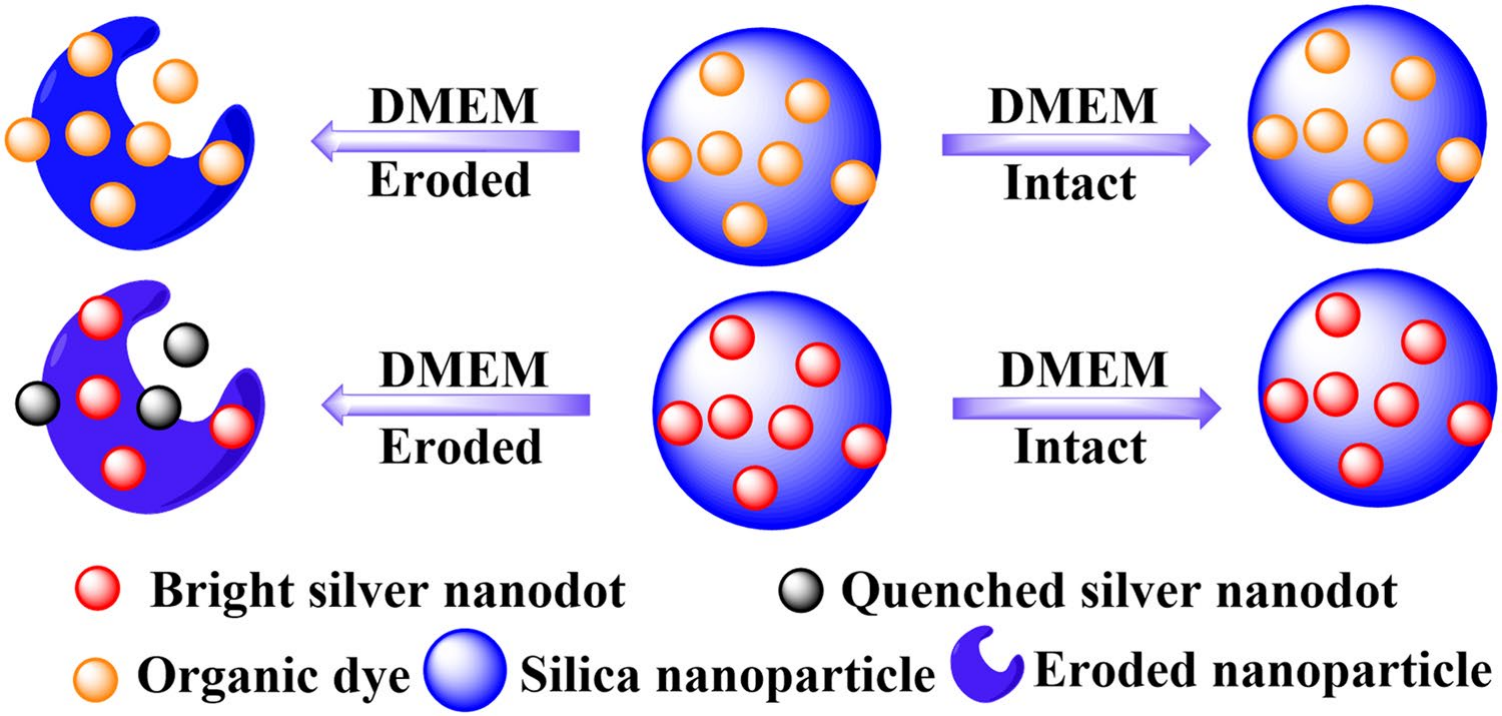
Schematic showing that silver nanodots-encapsulated in silica reveals the erosion of silica.

Luminescent noble metal-based clusters have attracted huge attention in the last decade. Their excellent photophysical properties and sensitivity to surrounding environments lead to the massive applications of these clusters in bioimaging and sensing^26–28^. Luminescent silver nanodots (stable ligand-protected silver clusters) are unique in their detection of silica stability simply with a fluorometer^29–32^. These ligand-protected silver clusters are stable in aqueous solution, but deteriorate in the presence of strong binding groups for silver, such as chloride in phosphate buffered saline (PBS) and organic molecules in cellular media^33,34^. We have developed methods to directly encapsulate silver nanodots in silica nanoparticles^35^. When encapsulated in silica nanoparticles, luminescent silver nanodots should be stable in any medium. However, the erosion of silica will lead to the exposure of silver nanodots to the medium, resulting in quenching of their photoluminescence by the medium. The conventional methods such as TEM and EDS to investigate the silica stability show images of the silica morphology and components. However, the preparation, imaging, and analysis of the samples are time-consuming, particularly when there are tens of samples to be examined. On the contrary, when we encapsulated a special fluorophore into the silica nanoparticles, the emission intensity of the fluorophore will indicate the status of silica. This strategy surpasses any possible methods utilizing ordinary organic dye-encapsulating silica nanoparticles to study the silica stability because the released organic dyes from silica are still fluorescent, indicating no direct sign of silica degradation (Fig. 1). By measuring the photoresponse of silver nanodot-encapsulating silica nanoparticles to a variety of ingredients in a cellular medium, we observed that the amino group containing components were primarily responsible for the accelerated degradation in a biological medium.

## Results

**Figure 2:**
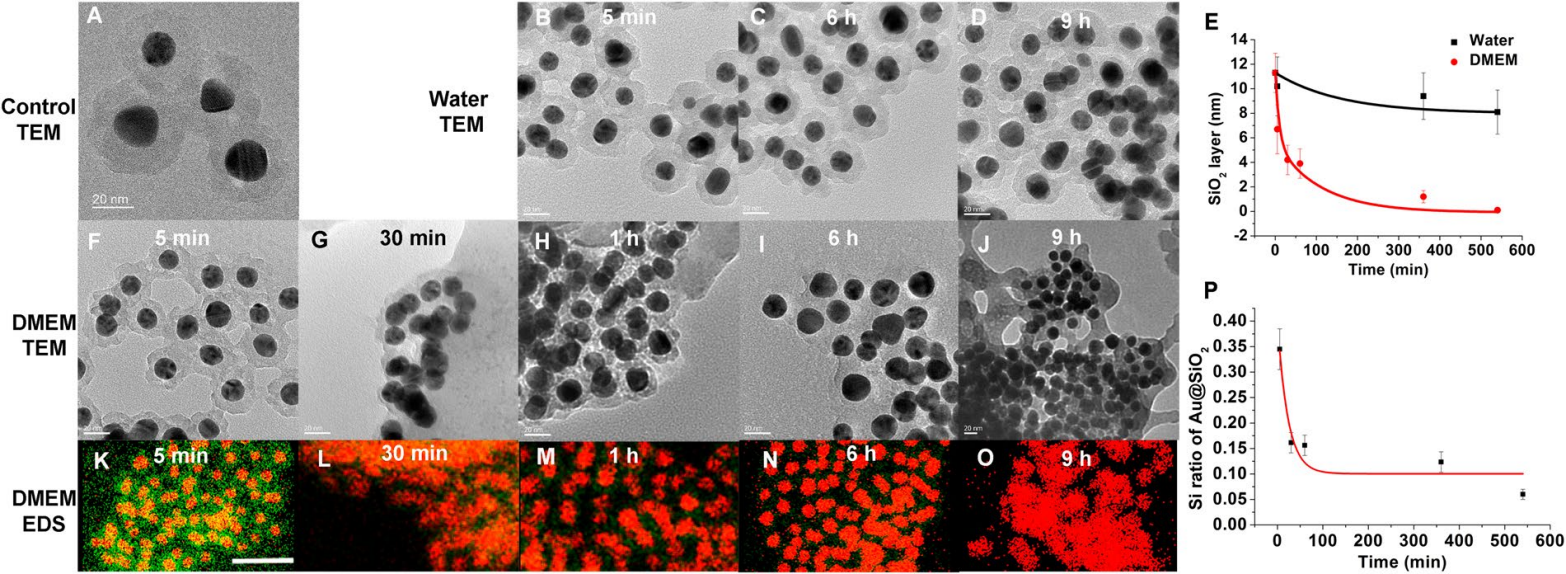
*In vitro* stability of silica shells. (**A**–**D**) TEM images of Au@SiO_2_ (**A**, control) and in water at 5 min, 6 h and 9 h, respectively (**B**–**D**). Scale bars, 20 nm. (**E**) Plots of the thickness of silica shells versus time in water (black) and DMEM (red), measured in TEM images. The error bars indicate standard error. The *p* value in each statistical analysis was less than 0.001. (**F**–**J**) TEM images of Au@SiO_2_ in DMEM at 5 min, 30 min, 1 h, 6 h and 9 h, respectively. Scale bars, 20 nm. (**K**–**O**) EDS images of Au@SiO_2_ in DMEM at 5 min, 30 min, 1 h, 6 h and 9 h, respectively. The images were merged from signals of gold in pseudocolor red and that of silicon in pseudocolor green. Scale bars, 100 nm. (**P**) Plots of the EDS intensity of silicon versus time in DMEM, measured from EDS images.

Nanomaterials for drug delivery are usually first examined *in vitro* with the cell lines of interest. Cell culture medium is the primary environment for such nanomaterials. We, therefore, investigated the interaction between silica coated gold nanoparticles (Au@SiO_2_) and a popular medium, Dulbecco’s Modified Eagle’s medium (DMEM), in which the gold core was used as a reference to locate the center of the Au@SiO_2_ particles in TEM images. The commercially available Au@SiO_2_ exhibited a core size of approximately 20 nm and a silica shell of 11.3 ± 1.6 nm (Fig. 2A). The solubility of silica in water depends on the pH of the solution and the structure of the silica^36,37^. As expected, the silica dissolved slowly in fresh deionized water. The silica layer became thinner, reaching 8.1 ± 1.8 nm in 9 h with a thickness shrinking constant of (1.5 ± 0.4) × 10^−8^ nm^−2^ s^−1^ when calculated following the shrinking sphere model (Fig. 2B–E and Supporting Information Figure S3)^38^. The degradation of silica was significantly accelerated in the presence of DMEM (Fig. 2F–J) at 37 °C. TEM images indicate that cracks or dents in the silica layer appeared within a few minutes of DMEM incubation (Fig. 2F) and these dents became dominant within an hour (Fig. 2H). The silica shell disappeared and spread nearby the gold nanoparticles after 9 h incubation (Fig. 2J). The degradation profile of the silica shell obtained from TEM images showed a thickness shrinking constant of (2.4 ± 0.2) × 10^−6^ nm^−2^ s^−1^. Note that there was a dark background in some TEM images. This can be ascribed to the residues of DMEM such as organic molecules and salts that either formed a thin film (Fig. 2G) or deposited in the area surrounding the nanoparticles (Fig. 2J). We have paid close attention to distinguish the boundaries of the nanoparticles when measuring the thickness of the silica layer. Therefore, energy dispersive X-ray spectroscopy (EDS) was applied to quantitatively measure the degradation of the silica shell (Fig. 2K–P and Supporting Information Figure S4), resulting in a silica degradation constant of (6.0 ± 0.8) × 10^−7^ nm^−2^ s^−1^ (Fig. 2P). The supplementation of foetal bovine serum (FBS, 10%) in the DMEM slowed the degradation of silica compared to DMEM, likely due to the adsorption of proteins on the silica surface (Supporting Information Figure S5)^39^.

**Figure 3:**
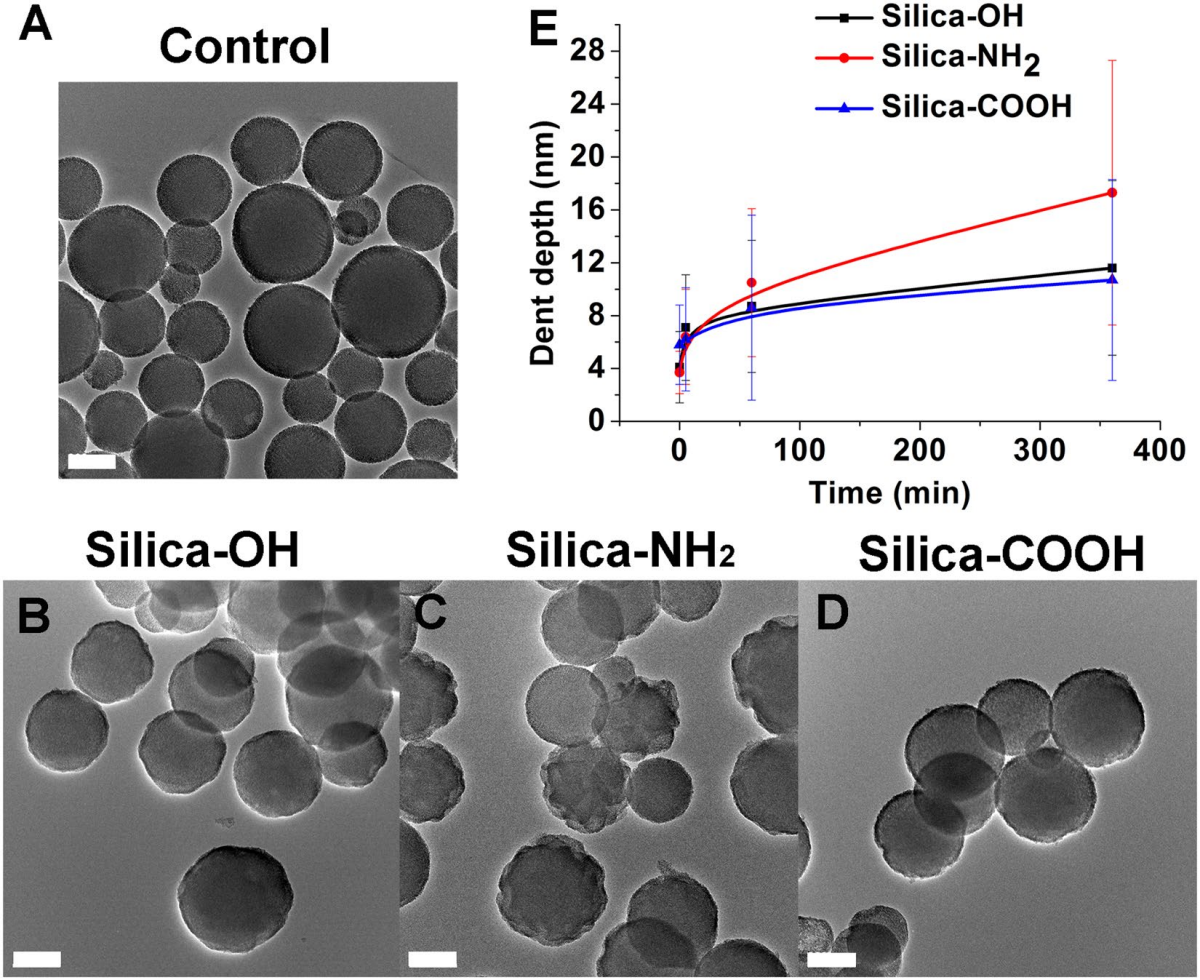
Degradation of mesoporous silica nanoparticles in DMEM. (**A**) Representative TEM image of mesoporous silica nanoparticles. (**B**–**D**) TEM images of original (**B**), amino group-modified (**C**) and carboxylic group-modified (**D**) MSNs in DMEM at 9 h. Scale bars, 200 nm. (**E**) Plots of the dent depth of MSNs versus time in DMEM. The error bars indicate standard error. The *p* value in each statistical analysis was less than 0.001.

Similar degradation was also observed among mesoporous silica nanoparticles (MSNs). The commercially available MSNs (approximately 0.2 μm in diameter with a 4 nm pore size) did not show a narrow size distribution (Fig. 3A), which made it difficult to monitor the decrease in diameter of silica nanoparticles. However, dents on the surface of MSNs appeared upon incubation with DMEM at 37 °C. The longer the incubation time, the deeper the dents (Fig. 3B and Supporting Information Figure S2 and Figure S6). In addition, surface modification influenced the degradation rate. Propylcarboxylic acid functionalized MSNs exhibited a similar degradation profile as that of the non-modified, whereas propylamine functionalized MSNs degraded with more and deeper dents (Fig. 3C,D).

**Figure 4:**
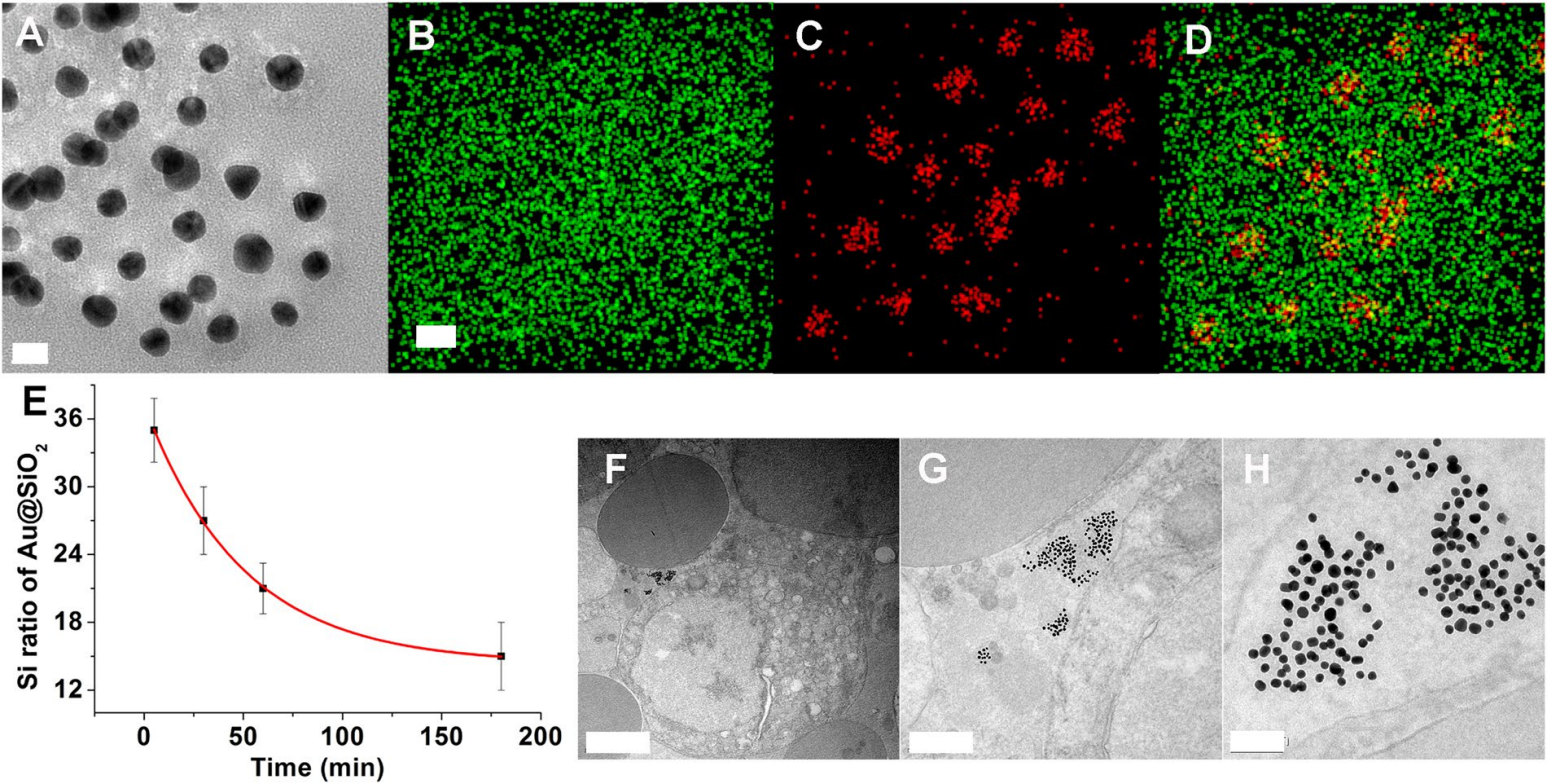
*Ex vivo* stability of silica in sheep blood. (**A**–**D**) TEM (**A**) and EDS (**B**–**D**) images of Au@SiO_2_, incubated in blood for 5 min. Scale bars, 20 nm. (**E**) Plots of the EDS intensity of silicon versus time, measured from EDS images. (**F**–**H**) TEM images of Au@SiO_2_ in sheep blood at various magnification. Scale bars, 2 μm, 0.5 μm and 100 nm for (**F**,**G** and **H**), respectively.

The importance of the stability of the silica in the core-silica shell structure prompted us to investigate the stability of silica *ex vivo*. Au@SiO_2_ nanoparticles were incubated in sheep blood at 37 °C and separated from the blood before being examined by TEM and EDS. Similar to what observed in DMEM, the silica shell degraded in blood, but exhibited a crumbled surface, as shown by the uneven color surrounding the gold core in (Fig. 4A) and Supporting Information Figure S7. A wider spread of silica away from the gold core was also detected in EDS images (Fig. 4B–D). This may suggest that cracks had formed before the harvest of the nanoparticles for electron microscope examination. The fragile shell collapsed and spread to the area surrounded the gold core. Fitting of EDS images yielded a silica shell degradation constant of (2.7 ± 0.1) × 10^−7^ nm^−2^ s^−1^ (Fig. 4E and Supporting Information Figure S8), which is slower than the erosion of silica in DMEM. This may be ascribed to a lower concentration of small organic molecules in blood compared to that in DMEM. To preclude any possibility of damage to the fragile silica shell occurring during nanoparticle separation by centrifugation, blood incubated with Au@SiO_2_ for 9 h was fixed before TEM examination. TEM images showed that the nanoparticles stayed between blood cells, with no sign of silica shell remaining (Fig. 4F–H). This further confirmed that the silica shells were eroded by ingredients of biomedia.

**Figure 5:**
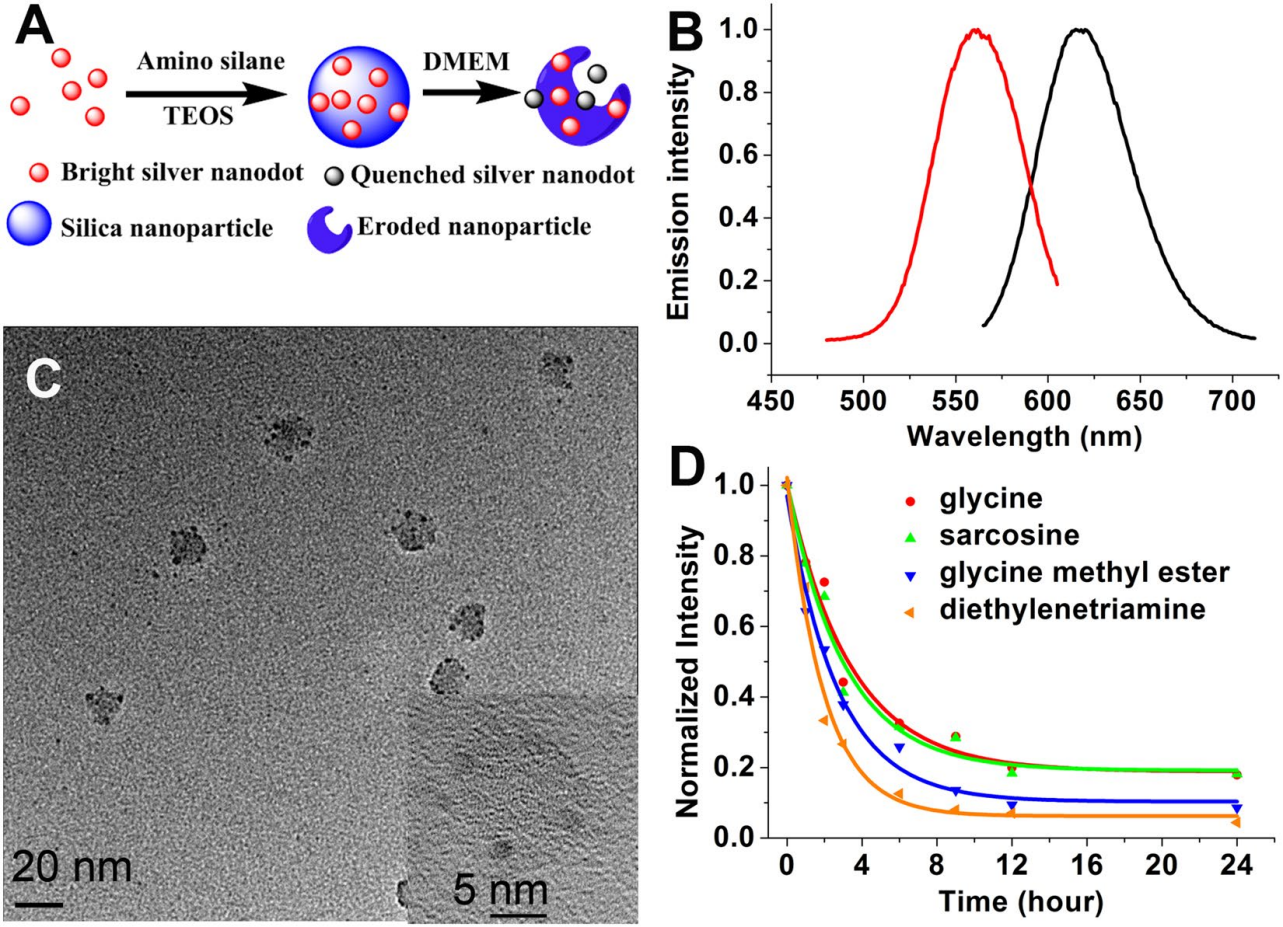
Detection of silica degradation with luminescent silver nanodots. (**A**) Schematic showing the encapsulation of silver nanodots in silica nanoparticles and their optical detection of silica degradation. (**B**) Emission (black) and excitation (red) spectra of the 615-emitter. (**C**) TEM image of silver nanodot-encapsulating silica nanoparticles (AgND@SiO_2_). Scale bar, 20 nm. Inset showing a high resolution image of the above nanoparticles. Scale bar, 5 nm. (**D**) Normalized luminescence intensity decay of the above AgND@SiO_2_ in the presence of glycine (red), sarcosine (green), glycine methyl ester (blue) and diethylenetriamine (orange).

To avoid elaborate TEM examinations and to study the main cause of the erosion in a simple and economical manner, luminescent silver nanodots (AgNDs) were used to monitor the degradation of silica by the ingredients of a cell culture medium. In our strategy, the bright nanodots will be quenched upon silica erosion (Fig. 5A). We successfully encapsulated single-stranded DNA stabilized silver nanodots (615 nm emission @ 560 nm excitation, black spots in TEM images) in silica nanoparticles (AgND@SiO_2_) with a mean diameter of 7 nm (Fig. 5B,C and Supporting Information Figure S9). Silver nanodots originally appeared light black spots as shown in a TEM image (Figure S10). When encapsulated in silica, they aggregated and appeared as clear dark spots. The above AgND@SiO_2_ nanoparticles were incubated in an aqueous solution consisting of a specific ingredient from DMEM, and the decay in luminescence intensity was used as a comparison of silica degradation rates. Results indicate that a specific amino acid, such as tyrosine, threonine, or glycine, barely accelerated the degradation rate of silica, likely because amino acids are present as zwitterions in water (Fig. 5D and Supporting Information Table S1). N-methylglycine (sarcosine), where the amino group is protected while the carboxylic acid is free, did not change the decay profile. Carboxylic acids, such as folic acid, did not change the profile either. This is in agreement with the fact that carboxylate ions interact weakly with silica and did not exert much influence on the degradation of silica^40^. Hydroxyl-rich compounds, such as riboflavin and glucose, also had little impact on the degradation of silica. However, the presence of the amino group in glycine methyl ester increased the silica degradation rate, which was further observed upon the addition of the amino group-rich compound diethylenetriamine. In the absence of silica, the silver nanodot decayed much faster with a survival half-life of less than 0.5 ± 0.2 h.

**Figure 6:**
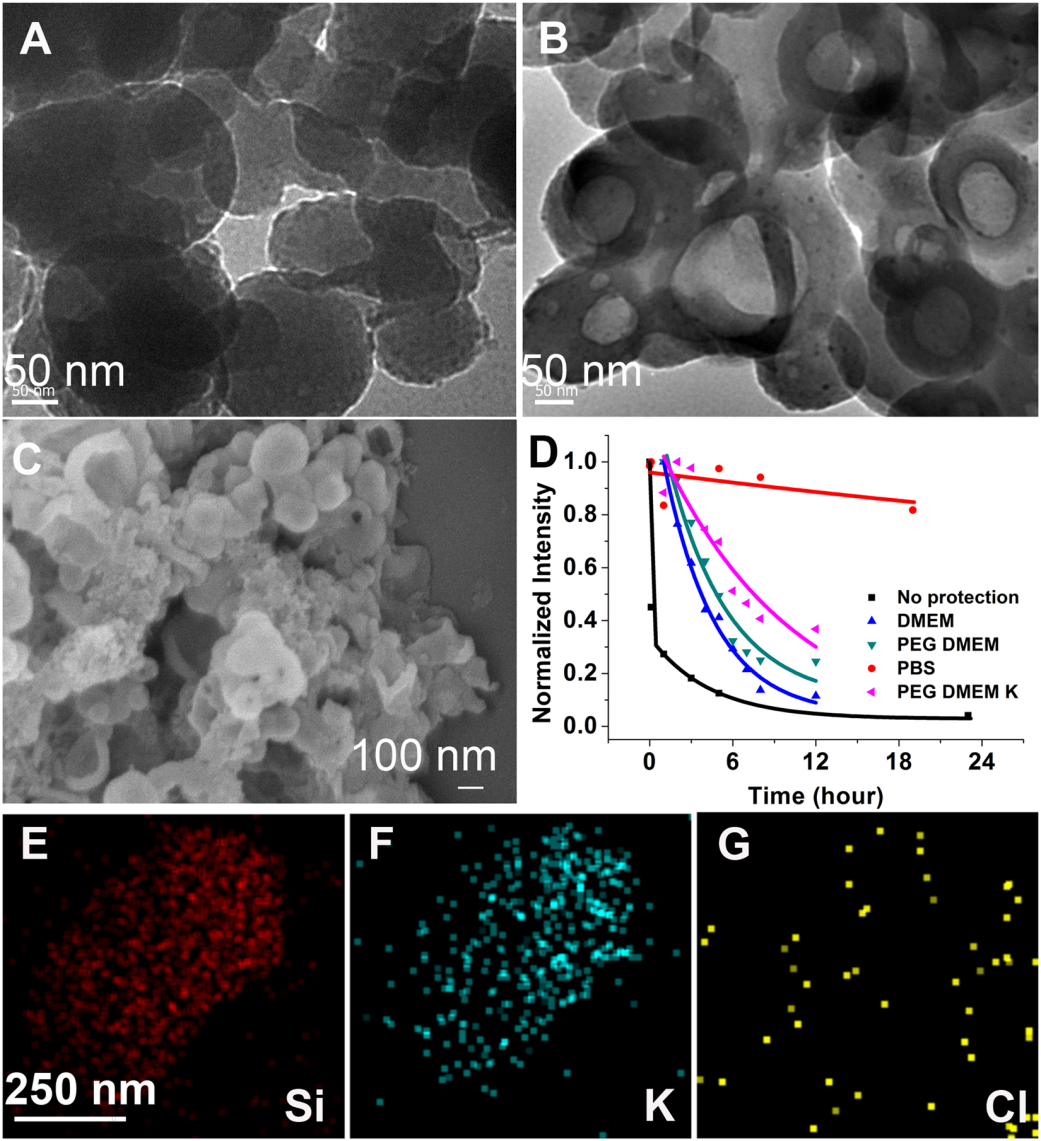
Improved silica stability. (**A + B**) TEM images of larger AgND@SiO_2_ after 5 min (**A**) and 9 h (**B**) incubation in DMEM. Scale bars, 50 nm. (**C**) SEM image of the sample in (**B**). Scale bar, 100 nm. (**D**) Comparison of luminescence intensity decay of silver nanodots under various conditions. (**E**–**G**) EDS images of the above AgND@SiO_2_ in PBS showing the signal of silicon (**E**), potassium (**F**) and chlorine (**G**). Scale bars, 250 nm.

Although increasing the thickness of the silica layer, for example to a 105 ± 35 nm, extended the degradation time of silica and improved silver nanodot stability in DMEM, the layer of silica in the above AgND@SiO_2_ slowed, but cannot prevent the quenching of luminescence of silver nanodots in DMEM. The interior of silica nanoparticles became hollow in DMEM after 9 h incubation, leading to formation of spherical shells as shown in the TEM and SEM images (Fig. 6A–C and Supporting Information Figure S11 (size distribution) and S12). The hollow interior resulted from DMEM etching via dents on the silica surface, whereas the spherical shell can be ascribed to the formation of a stable apatite-like surface^21^. Silver nanodots encapsulated in the above large silica nanoparticles demonstrated a survival half-life of 2.6 ± 0.3 h in DMEM, significantly longer than that of unprotected silver nanodots in DMEM (0.07 ± 0.02 h). To reduce the degradation of silica in medium, PEG (mPEG5K-Silane, average M_n_ 5,000) was used to modify the silica surface. A surface coating of this nature extended its half-life to 3.0 ± 0.2 h (Fig. 6D, Figures S13 and S14). Surprisingly, the survival half-life of silica nanoparticles in PBS increased significantly to 57 ± 10 h, even longer than that in water. This suggested that components in PBS dramatically stabilized the silica surface. EDS images of the above stabilized silica nanoparticles showed that, other than phosphate, chloride and sodium, potassium was highly colocalized with the silicon element (Fig. 6E–G and Supporting Information Figure S15). We, therefore, tested the addition of additional potassium ions (K_2_HPO_4_, 30 mM) into DMEM and observed significantly improved stability of silica with a survival half-life of 6.7 ± 0.5 h in DMEM (PEG DMEM K in Fig. 6D).

## Discussion

The fast degradation of silica in DMEM raised a caution flag regarding the silica-coated nanoparticles. The popularity of DMEM implied that it is being used in many *in vitro* experiments. However, our results indicate that the surface silica may be eroded by the ingredients of DMEM, leading to the exposure of core materials. This may cause misinterpretation of experiments regarding activity of silica-protected nanomaterials. The chemical and physical properties of the above nanomaterials may be different from what is expected since surface chemistry specifically determines the properties of nanoparticles^41^. The erosion of the silica likely starts with a weak point of the surface. It is important to note that the thickness of the silica layer of Au@SiO_2_ nanoparticles was measured from the thinnest part of the silica layer in a particular TEM image (Supporting Information Figure S1). The faster degradation rate obtained from the TEM images compared to that from EDS suggests that dents formed deeper and closer to the gold core before silica detached from the outer shell, as shown in Fig. 2H. Such dents or cracks were proposed in a previous report to explain the excitation of oxygen by silica-encapsulated semiconductor quantum dots^12^.

Amino groups appear to play an important role in the erosion of silica. Note that propylamine-modified mesoporous silica nanoparticles also demonstrated a faster degradation than propylcarboxylic acid modified nanoparticles. The aggravated etching by amino groups may be ascribed to the fact that amino-rich compounds can stabilize monomeric units of silica and slow down silicic acid polycondensation^42^. The electrostatic attraction between the negatively charged silica nanoparticles and the protonated amino groups facilitates the bonding between nitrogen and surface silica and then enhances the dissolution of silica nanoparticles. The amino-rich groups in DMEM synergistically etched the silica surface, resulting in a significant increase in the degradation rate of silica.

The stability of silica depends on the silica structure as well. The mesoporous silica nanoparticles are prepared at a higher temperature compared to the core-silica shell nanoparticles. There is a higher level of polymerization in the mesoporous silica nanoparticles, which show a better stability. A recent report indicates that the presence of possible porous structures in the silica layer of the core-shell nanostructures^43^. Potentially the porosity of the silica nanoparticles will influence the stability of the silica. The larger surface area due to a larger porosity may lead to more vigorous erosion from the surrounding materials. In addition, the encapsulation of external molecules in the silica networks may also cause the disintegration of the silica and induce faster erosion. The silver nanodot-encapsulating silica nanoparticles degraded much more severely than the mesoporous silica nanoparticles, likely because intercalation of foreign substances weakened the strength of the silica network. Therefore, this facilitated the formation of cracks/dents in the inner core of silica, resulting in faster degradation. Moreover, the silica may aggregate or agglomerate in the aqueous solution. Sometimes the silica nanoparticles appeared separated from each other but sometimes agglomerated in the TEM images. The agglomeration was likely due to the preparation of the TEM samples as the concentration of the silica nanoparticles was too high. However, it was also possible that the silica nanoparticle aggregated since there was no steric protection of the silica on its surface. The DLS measurements on some of the silica nanoparticles (Figure S14) showed that there were three peaks in the DLS spectrum of the silica nanoparticles embedded with silver nanodots. The smallest one was about 100 nm in hydrodynamic diameter, which can be assigned to the single silica nanoparticle. There was a peak centered at 1000 nanometer. This can be the hydrodynamic diameter of the aggregation or agglomeration of the silica nanoparticles. Therefore, we cannot exclude the possibility that the silica agglomerated/aggregated in the aqueous solution.

Large protection groups at the silica surface do not increase the stability of silica. Even though the PEG-modified AgND@SiO_2_ exhibited strong infrared signals indicative of PEG and a significantly increased hydrodynamic radius (Figures S13 and S14), we cannot exclude the possibility that the surface modification of the silica surface had not been perfect. However, the limited improvement of its stability was likely due to the resulting highly heterogeneous surface after modification^44^. It is possible that surface coatings cannot fully cover the surface, while small molecules can access the silica surface. Surprisingly, small ionic species may offer a better solution to improve the stability of silica. The negatively-charged nature of silica nanoparticles allows cations to adsorb onto the nanoparticle surface. It is not evident why silica nanoparticles showed a preference for potassium ions over sodium ions. This may be due to a specific arrangement of charges and defects on the surface, just like crown ether, which matched the size of potassium ions and significantly increased the affinity for potassium^45^. It has been reported that potassium ions increase the mechanical strength of silica glass^46^, which was also accompanied by decreased solubility of silica in a salt solution of PBS, resulting in improved silica stability^47^. In addition, the formation of a potassium layer may form a shell to shield the silica from hydrolysis. The outstanding stability of silica nanoparticles in PBS was also supported in previous *in vitro* research^48^.

The environments inside the cells and in the DMEM are different. The lower pH of endosomes/lysosomes suggests that the silica nanoparticles degrade slowly once these nanoparticles enter the endosomes of the cell via endocytosis. Once, if there is any chance, the nanoparticles enter the cytosol of the cells, the increased pH accelerates the degradation of the silica becomes more severely. Even though silica may be vulnerable to certain biological media, we can tune the size, silica structure, surface chemistry and environments of silica nanoparticles to temporally and spatially control the degradation of silica, which may assist in the development and fabrication of silica-based drug delivery systems.

In summary, we have demonstrated that luminescent silver nanodots can be used as an indicator for the erosion of silica. The degradation of the silica in silver nanodot-encapsulating silica nanoparticles leads to the exposure and subsequent quenching of silver nanodots, which reflects the stability of the surface silica. The erosion of silica strongly depended on the biological medium surrounding it. Though silica nanoparticles showed extraordinary stability in PBS, a synergistic etching of silica by medium components, particularly the amino-rich compounds in cell culture medium as well as blood, deteriorated the silica layers. Such surface degradations were accompanied by a faster growth of cracks/dents approaching the inner core, resulting in the detachment and crumbling of surface silica. The rapid erosion of silica in various biological media suggests that the survival time of silica nanostructures is a critical factor when assessing fabricating silica-based drug delivery systems. Meanwhile, this degradation also enables us to design a system with spatial and temporal control of drug release from silica nanostructures.

## Methods

### Au@SiO_2_ degradation test in medium

Au@SiO_2_ nanoparticles (obtained from Sigma-Aldrich, 1 OD at 520 nm) were concentrated by centrifugation and re-suspended in 1 mL of fresh de-ionized water, PBS, DMEM, DMEM supplemented with 10% FBS, and sheep blood, respectively. After 5 min, 30 min, 1 h, 3 h, 6 h and 9 h incubation in a CO_2_ incubator (5% CO_2_, 37 °C), samples were centrifuged at 16,000 rpm to collect Au@SiO_2_. The resulting nanoparticles were imaged with TEM and EDS.

### Au@SiO_2_ degradation calculations

The coating of silica on the surface of gold nanoparticles was reasonably even, as shown in Fig. 2A. However, the erosion of the silica layer was not even. Some part of the silica shell dissolved faster, where it was most likely that the exposure of the core gold to the environment occurred in the first place. The thickness of the thinnest part of the silica layer on the surface of a gold nanoparticle was regarded as the thickness of the silica layer (as the arrow indicated in Supporting Information Figure S1). The boundary was determined by distinguishing the tightly bound, round disk that surrounding the gold core nanoparticle. The nanoparticles whose boundaries cannot be distinguished in the TEM images were not counted. The length was measured with ImageJ, in which a line was drawn across the distance of interest and /Analyze/Measure was clicked. The “length” in the result report was the thickness of the silica layer.

### Mesoporous silica nanoparticle (MSN) degradation tests in medium

Mesoporous silica nanoparticles (amino-modified, carboxylic acid-modified, and no modification, with a pore size of 4 nm, respectively; 0.1 mg each) were re-suspended in 1 mL of DMEM. After 5 min, 30 min, 1 h, 3 h, 6 h, and 9 h incubation in a CO_2_ incubator (5% CO_2_, 37 °C), samples were centrifuged at 16,000 rpm to collect the mesoporous silica nanoparticles. The resulting nanoparticles were imaged with TEM.

### Mesoporous silica nanoparticles degradation calculations

Any part of a nanoparticle experienced etching forces from the surrounding environment and the size of the nanoparticle shrank. However, the wide size distribution of the commercially available MSNs made it difficult to monitor the decrease in diameter of silica nanoparticles. Nevertheless, we observed severe erosion of the silica at various sites on the surface. We assumed that silica nanoparticles were spherical and therefore drew a circle around the particle to keep the ring match most of the edge of the particle. The degradation of mesoporous silica nanoparticles was measured from the dent depth of the silica layer from the circle in a TEM image (Supporting Information Figure S2). The distance was measured with ImageJ as described above.

### Silver nanodots preparation

ssDNA (CGCGCCCCCCCCCCCCCGCG, 50 μM) and silver ions were mixed at a DNA base/Ag^+^ ratio of 2:1 in water (2 mL), followed by the reduction with aqueous sodium borohydride (1 mg/mL, 50 μL). Silver nanodots were used as probes a day after chemical reduction of the mixture^49,50^.

### Silver nanodot-encapsulated silica nanoparticle preparation

In the early stage of AgND@SiO_2_ preparation, aqueous silver nanodots (125 μM based on ssDNA), NED (125 μM), and TEOS (1 mM) were mixed in aqueous solution and left at room temperature overnight. In the optimized protocol, aqueous silver nanodots (125 μM based on ssDNA) and NED (1 mM) were mixed in aqueous solution and left at room temperature for two hours, followed by addition of TEOS (16.7 mM). After 24 hour incubation, the solution was centrifuged at RCF 13,200 g to collect the silica nanoparticles.

### Surface modification of AgND@SiO_2_

Aqueous AgND@SiO_2_ solution was diluted with ethanol (water/ethanol 1:1). AgND@SiO_2_ was then collected by centrifugation and redispersed in ethanol. Extra silane mPEG5K-Silane (average M_n_ 5,000, 2 mg) was added into the new solution. The mixture was left at room temperature overnight, and collected by centrifugation.

### Data availability statement

The datasets generated during and/or analysed during the current study are available from the corresponding author on reasonable request.

## Electronic supplementary material

Supplementary Information
