# Supplementary material for: Silica nanoparticle stability in biological media revisited
Source: Sci Rep. 2018 Jan 9;8:185. doi: 10.1038/s41598-017-18502-8 (PMC5760698; doi:10.1038/s41598-017-18502-8)
Supplement: Supplementary file 1 — Supplementary Information [file 41598_2017_18502_MOESM1_ESM.pdf]

Silica Nanoparticle Stability in Biological Media Revisited

Seon-Ah Yang,[†] Sungmoon Choi,[†] Seon Mi Jeon, and Junhua Yu*

Department of Chemistry and Education， Seoul National University， 1 Gwanak-Ro, Gwanak-Gu, Seoul 08826, South Korea

junhua@snu.ac.kr

[†] These authors contributed equally to this work.

Chemicals. Silver nitrate (99.9999%), phosphate buffer saline (PBS), Dulbecco's Modified Eagle's Medium (DMEM), fetal bovine serum, sodium borohydride, tetraethyl orthosilicate (TEOS), N-[3-(Trimethoxysilyl)propyl]ethylenediamine (NED), mPEG5K-Silane (average M_n 5,000), mesoporous silica nanoparticles (200 nm in diameter, pore size 4 nm), propylcarboxylic acid functionalized mesoporous silica nanoparticles (200 nm in diameter, pore size 4 nm), propylamine functionalized mesoporous silica nanoparticles (200 nm in diameter, pore size 4 nm), glycine, sarcosine, glycine methyl ester, diethylenetriamine, riboflavin, tyrosine, threonine, folic Acid, glucose, potassium phosphate dibasic trihydrate, and Au@SiO_2 nanoparticles (20 nm gold core) were purchased from Sigma-Aldrich and used as received. Sheep blood was purchased from Thermo Scientific. ssDNAs were synthesized by Integrated DNA Technologies. Purified water (resistivity, 18.2 MΩ) was used.

Instruments. HRTEM images were obtained on a JEM 3010 high resolution transmission electron microscope. The size distribution of nanoparticles were obtained by counting the size of nanoparticles in multiple TEM images. Energy Dispersive Spectroscopy (EDS) results were obtained on a X-maxT (Oxford instruments). UV-Vis absorption spectra and emission spectra were obtained on a S-4100 (SCINCO) and QM-40 (Photon Technology International, Inc.), respectively. Microscopic emission images were obtained on an Olympus XI-81 microscope with a ×60 objective (NA 1.35) and an Andor Luca^{EM} S 658M camera. An Eppendorf cooling centrifuge 5415 R was used to collect silica nanoparticles. Samples at 37°C were stored in a CO2 incubator at 37°C (Thermo Fihser Scientific). Infrared spectra were obtained on a FT-IR Fourier Transform Infrared Spectrometer (TENSOR27, Bruker). SEM images were obtained on a Field Emission Scanning Electron Microscope (JSM-7800F Prime, JEOL). DLS was analyzed with a Dynamic Light Scattering Spectrophotometer (DLS-7000).

Degradation constant. The dissolution of the surface silica depends on the surface area and the number of surface molecules ($dM/dt = -k_{TEM}AM$, where A is the surface area, M the number of surface molecules, and k the degradation constant). Given that the radius of the nanoparticle is r and the molecular size of the silica unit is d_o, the above equation gives $\frac{d(4\pi r^2/d_o)}{dt} = -\boldsymbol{k4\pi r^2\, 4\pi r^2/d_o}$. The solution to the equation is $\boldsymbol{1/r^2 = 1/r_o{}^2 + kt}$. Fitting the curve in Figure 1e yield k_{TEM}. Similarly, the equation for the EDS analysis is $dV/dt = -k_{EDS}AV$, where A is the surface area, k_{EDS} the degradation constant, and V the volume of the nanoparticle. The decay of the silicon value in EDS analysis was considered to be the change in the silica volume. Monoexponential fit of the curve in Figure 1p yields a k that is a product of k_{EDS} and A. Its unit is $nm^{-2}\ s^{-1}$.

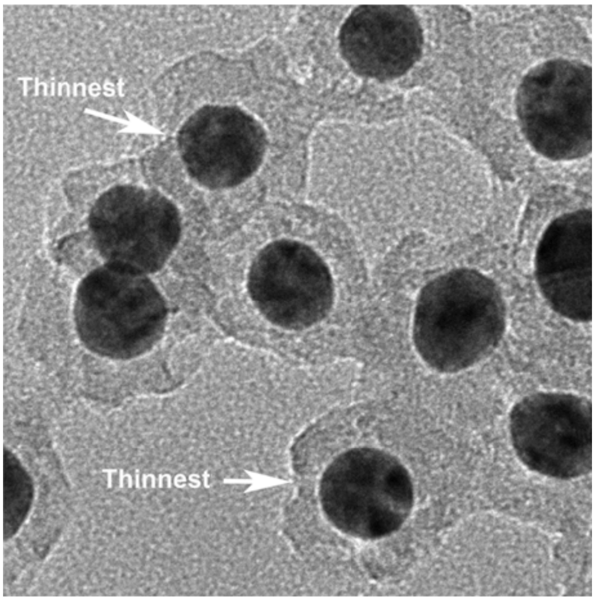

Figure S1. Method to measure the thickness of the silica shell of the Au@SiO_2 nanoparticles. The thinnest part was counted as the thickness of the eroded silica shell.

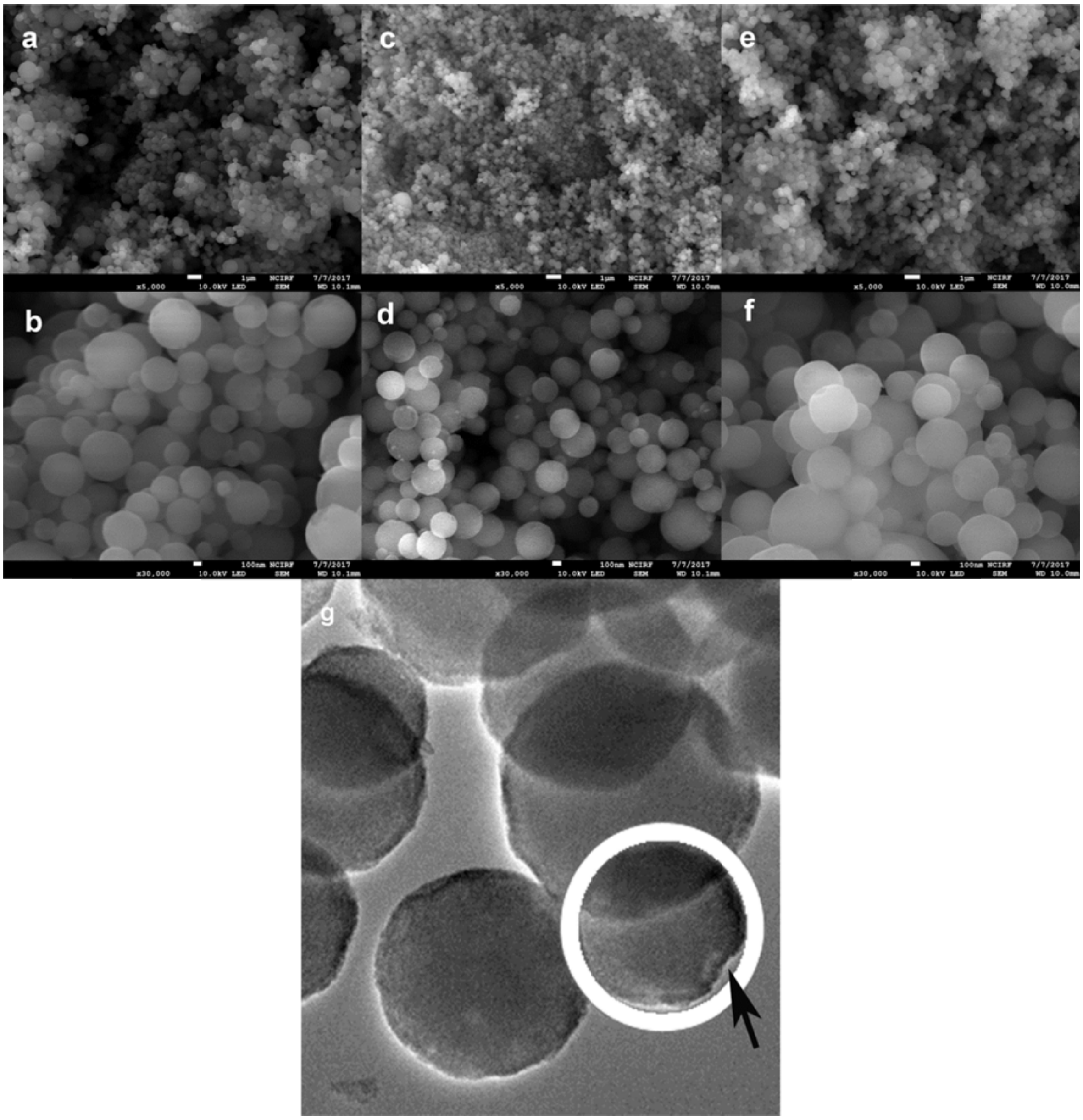

Figure S2. SEM images of MSNs and method to measure the depth of dents on the surface of mesoporous nanoparticles. a/b, c/d, and e/f are the SEM images of bare, carboxylic acid-modified, and amino-modified mesoporous silica nanoparticles, respectively. Scale bars, 1 μm for a, c, d, and 100 nm for b, d, e. The method to measure the depth of dents was illustrated in g. Any part of a nanoparticle that experienced etching forces from the surrounding environment and the size of the nanoparticle shrank. However, we observed severe erosion of the silica at various

sites on the surface. We assumed that silica nanoparticles were spherical and therefore drew a circle around the particle. The depth of the dent was counted as the degree of erosion.

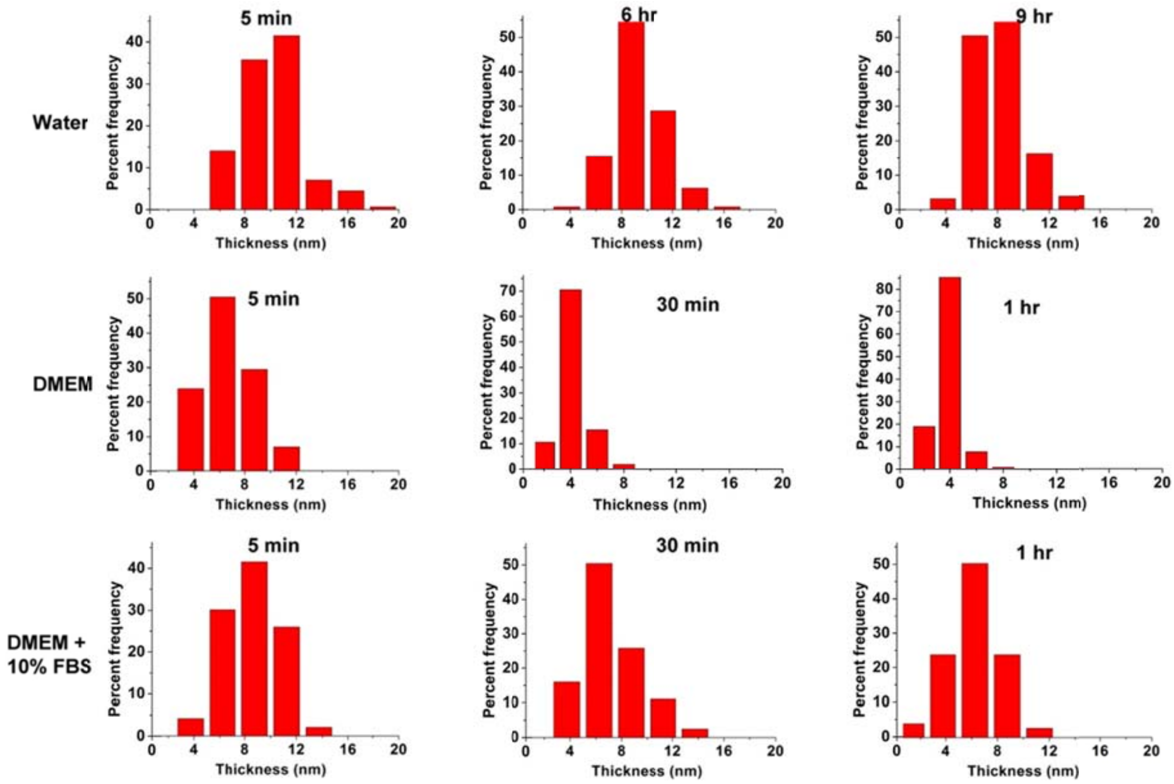

Figure S3. Size distribution of the thickness of SiO_2 layer in Au@SiO_2. Au@SiO_2 nanoparticles were incubated in water, DMEM and DMEM supplemented with 10% FBS for various time points and examined with TEM.

5 min

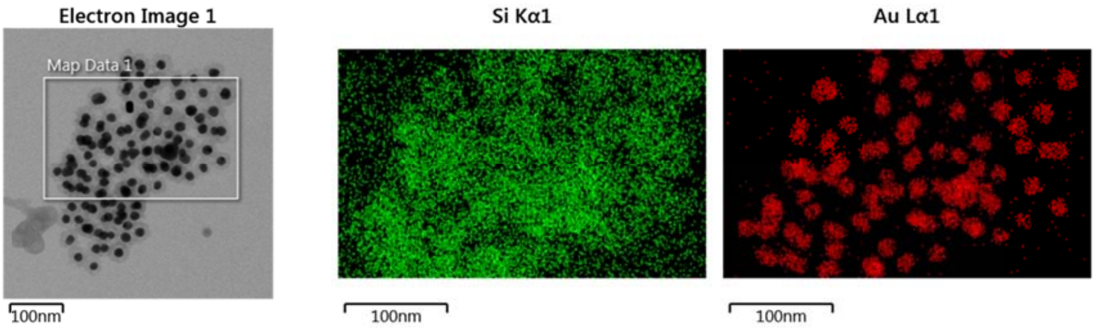

30 min

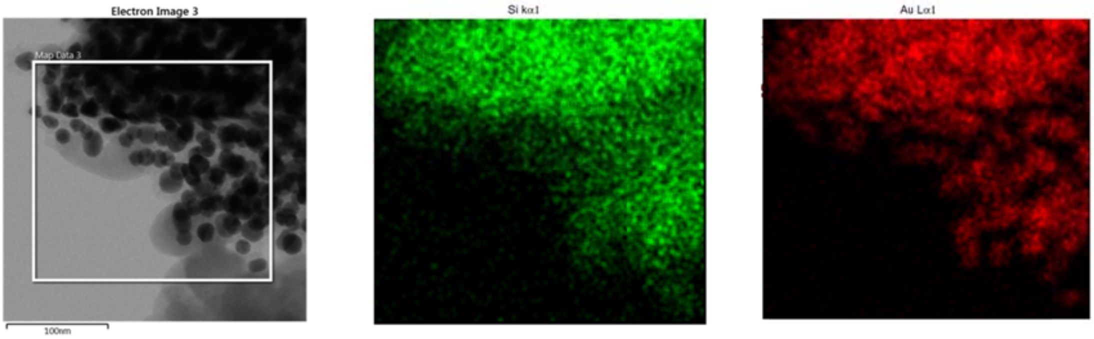

1 h

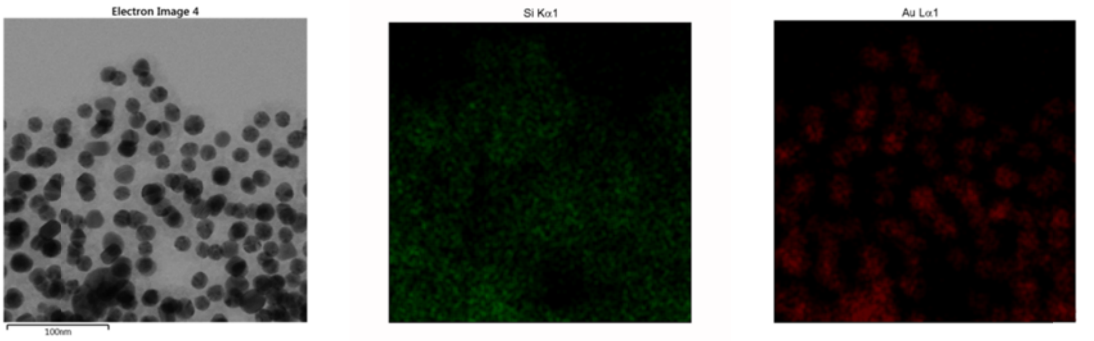

6 h

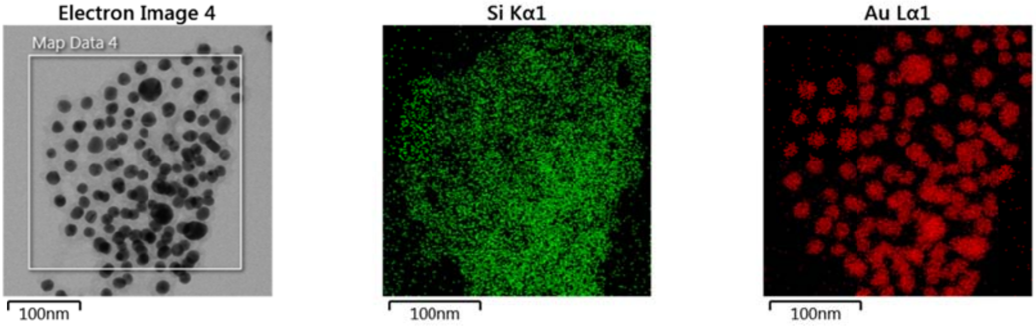

9 h

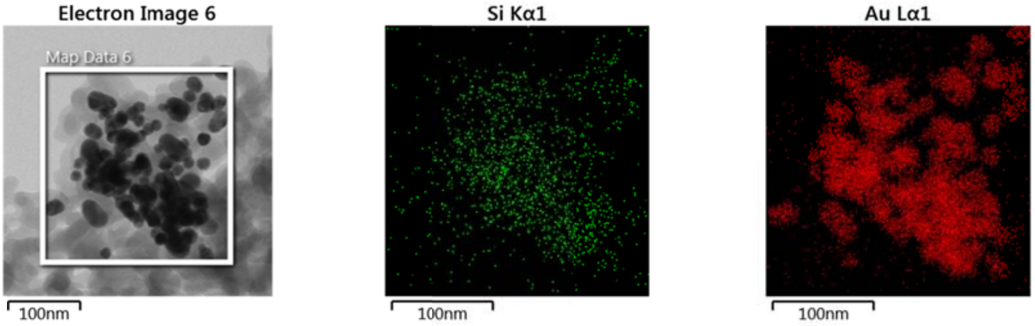

Figure S4. EDS measurements of silica coated gold nanoparticles at varied DMEM incubation times. Au@SiO_2 nanoparticles were incubated in DMEM for various times and examined with EDS. The left bright and white pictures show TEM images of the examined area. The middle images in pseudocolor green show EDS images of silicon and the right pseudocolor red show that of gold. Scale bars, 100 nm.

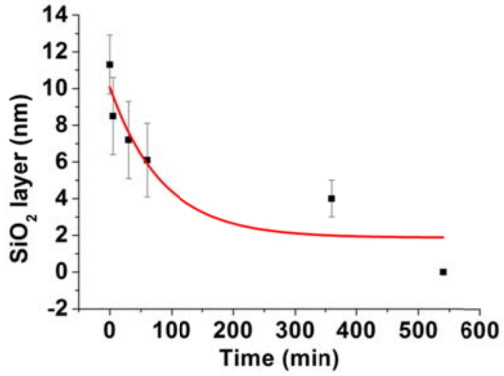

Figure S5. Thickness of the silica shell of the Au@SiO_2 nanoparticles upon the incubation in DMEM supplemented with 10% FBS. Data were obtained from TEM images and fitted exponentially.

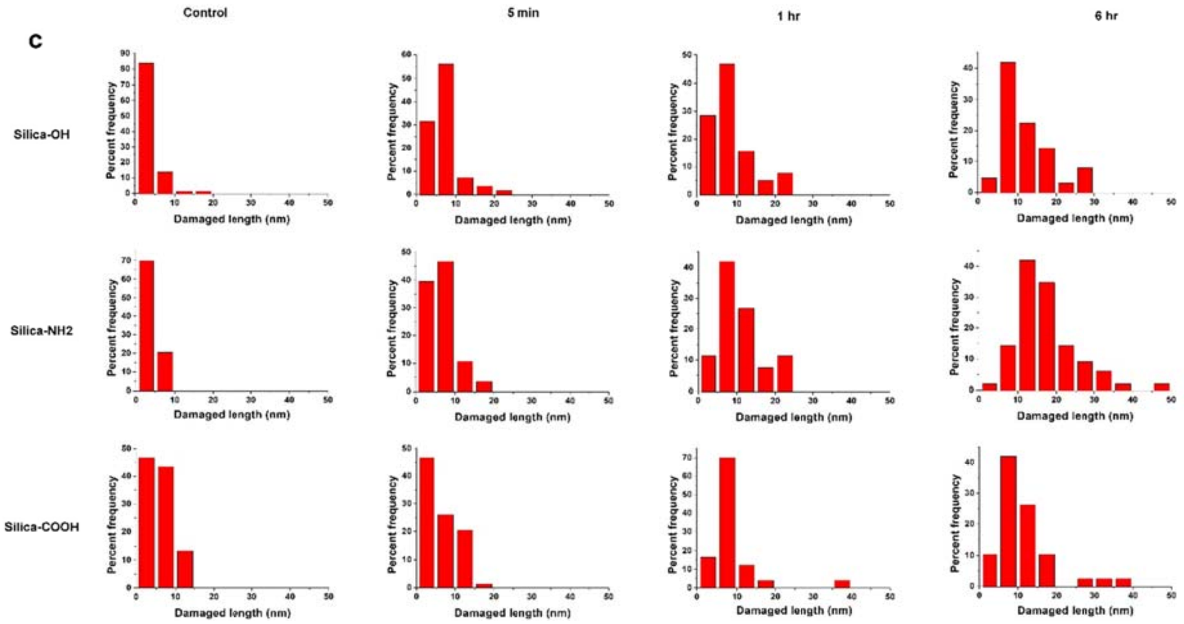

Figure S6. Size distribution of the dents in mesoporous silica with different surface modifications. Mesoporous silica nanoparticles (Silica-OH), propylamine-functionalized MSNs (Silica-NH_2), and propylcarboxylic acid-functionalized MSNs (Silica-COOH), were incubated in DMEM for various times and examined with TEM.

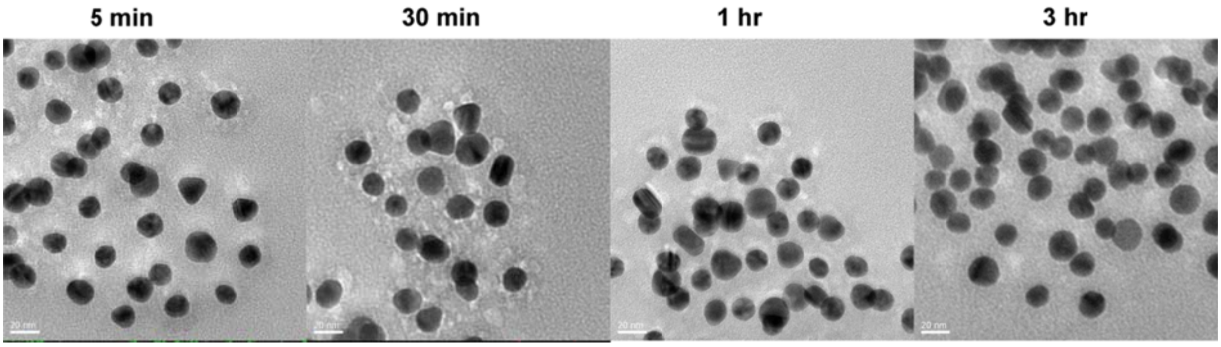

Figure S7. TEM measurements of silica coated gold nanoparticles at varied sheep blood incubation time. Scale bars, 20 nm.

5 min

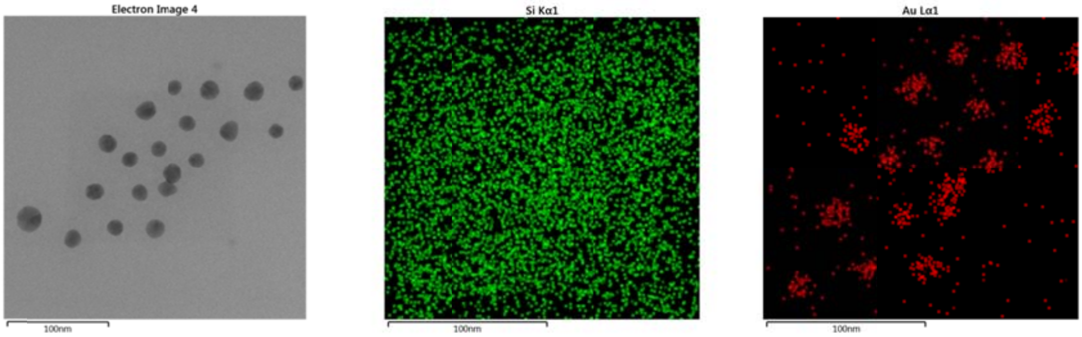

30 min

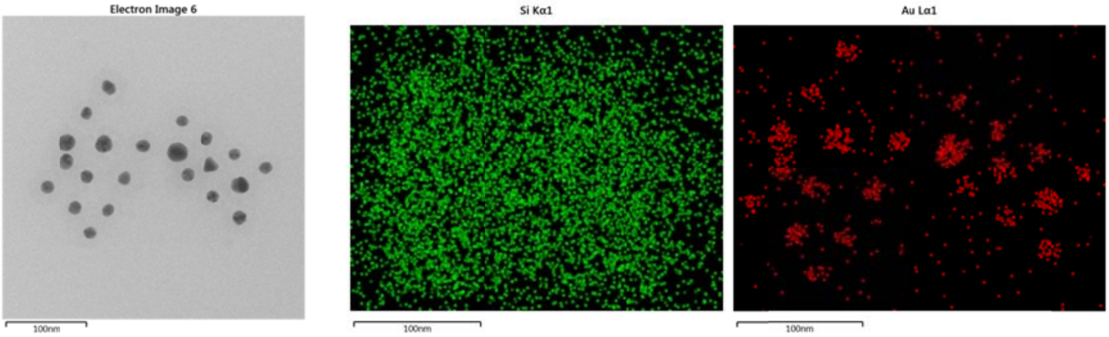

1 h

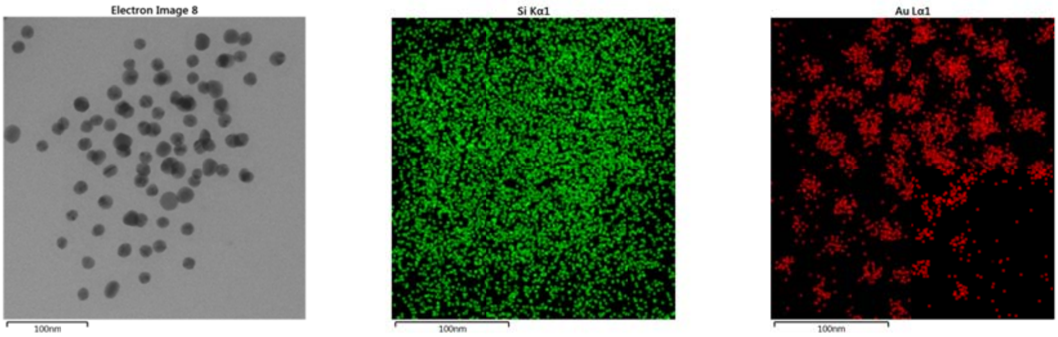

3 h

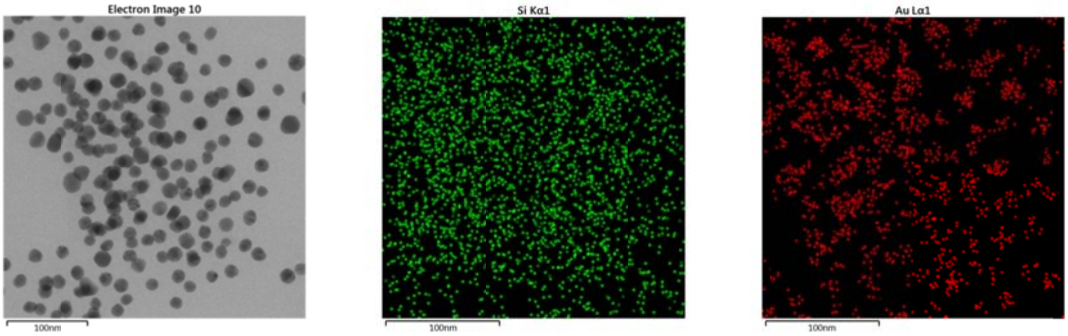

Figure S8. EDS measurements of silica coated gold nanoparticles at varied sheep blood incubation time. Au@SiO_2 nanoparticles were incubated in sheep blood for various times and examined with EDS. The left bright and white pictures showing TEM images of the examined area. The middle images in pseudocolor green showing EDS images of silicon and the right is pseudocolor red showing that of gold. Scale bars, 100 nm.

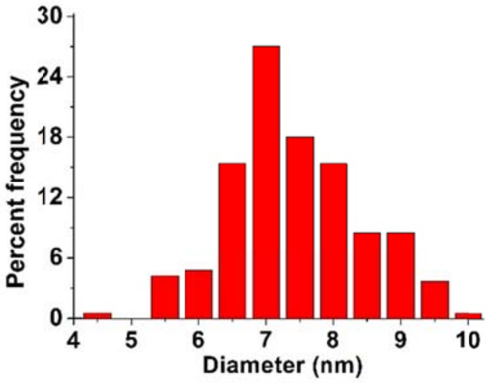

Figure S9. Size distribution of the silver-nanodot-encapsulating silica nanoparticles in Figure 4.

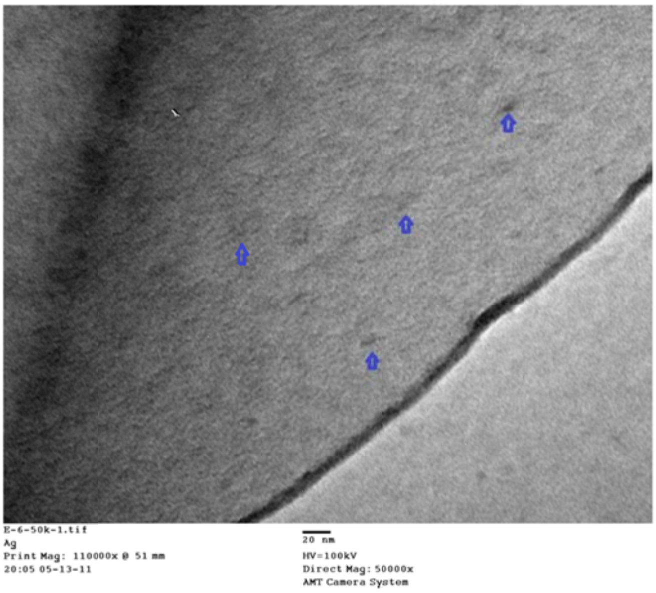

Figure S10. TEM image of silver nanodots.

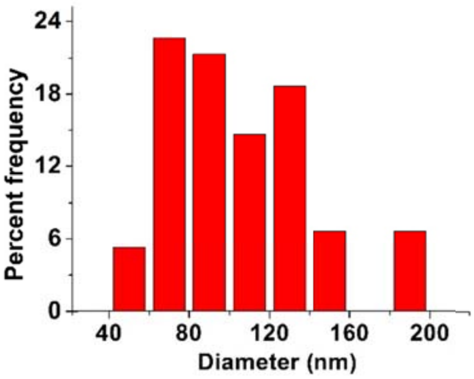

Figure S11. Size distribution of silver nanodot-encapsulating silica nanoparticles in Fig. 5a.

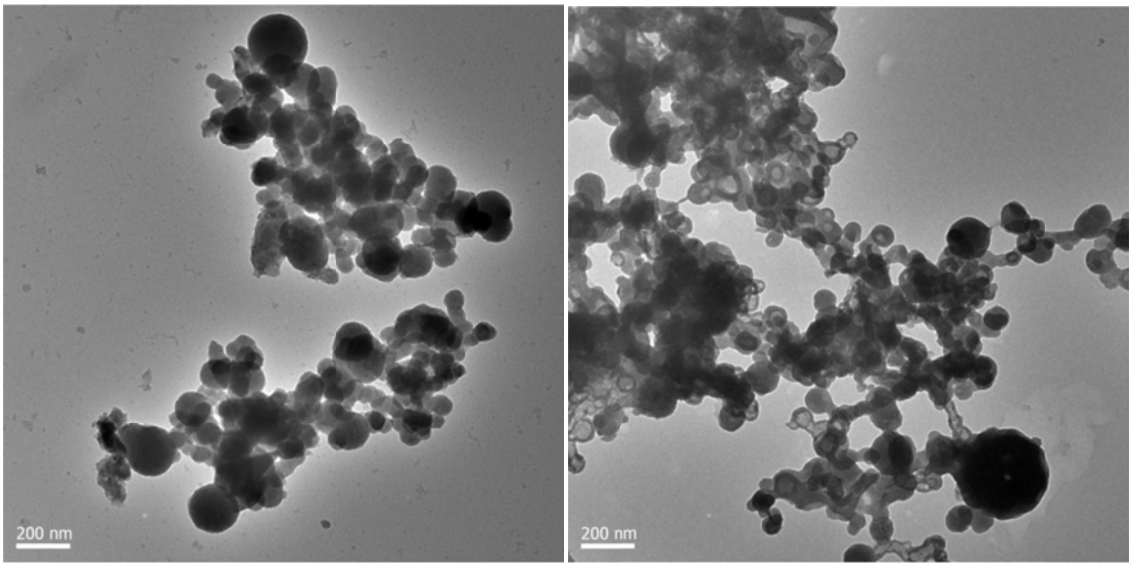

Figure S12. TEM images of silver nanodots-encapsulating silica nanoparticles. Before (left) and after (right) incubation in DMEM for 9 hours. Scale bars, 200 nm.

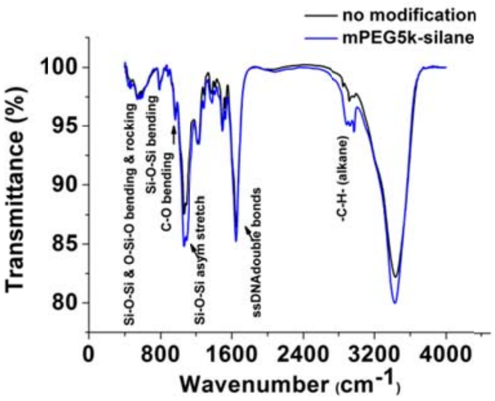

Figure S13. IR spectra of surface modified silica nanoparticles. Silver nanodot-encapsulating silica nanoparticles (no modification) were modified with PEG (mPEG5k-silane). Both nanoparticles were frozen dried and measured with infrared spectrometry.

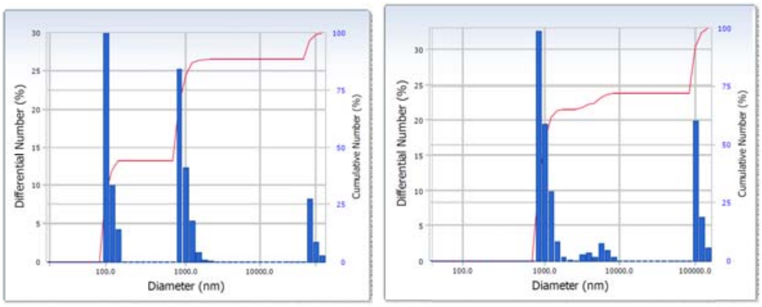

Figure S14. Dynamic light scattering spectra of surface modified silica nanoparticles. Left: no modification; right: mPEG5k-silane. DLS showing hydrodynamic radii of 110 ± 15 nm and 1150 ± 220 nm, respectively. Meanwhile, aggregates of these nanoparticles were observed in the same samples.

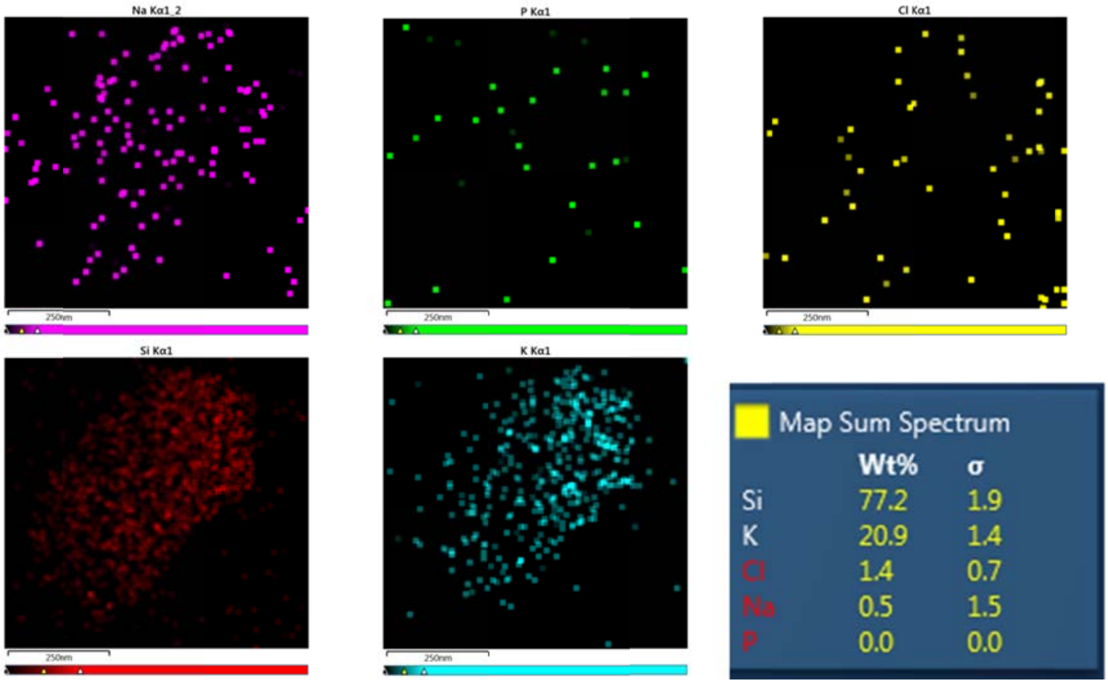

Figure S15. EDS of silica nanoparticles treated in PBS. Silica nanoparticles were incubated in PBS and separated by centrifugal ultrafiltration after three washes with DI water.

Table S1. Photoluminescence decay of silver nanodots in AgND@SiO_2 in the presence small organic molecules.

Compounds	Control	Riboflavin	Tyrosine	Threonine	Folic Acid	Glycine	N-methyl-glycine	Glycine methyl ester	Glucose	Diethylene-triamine
Luminescence Lifetime (h^{-1})	3.1	3.1	3.3	4.2	3.6	4.5	3.6	2.0	3.0	1.5
Standard deviation	0.4	0.5	0.5	0.4	0.4	0.6	0.6	0.1	0.7	0.4
